# Airborne particulate matter in public transport: a field study at major intersection points in Frankfurt am Main (Germany)

**DOI:** 10.1186/1745-6673-9-13

**Published:** 2014-04-09

**Authors:** Alexander Gerber, Julia Bohn, David A Groneberg, Johannes Schulze, Matthias Bundschuh

**Affiliations:** 1 Institute of Occupational Medicine, Social Medicine and Environmental Medicine, Goethe-University, Theodor-Stern-Kai 7, Haus 9b, 60590 Frankfurt am Main, Germany

## Abstract

**Background:**

Chronic particulate matter (PM) exposure is correlated to various health effects, even at low amounts. WHO has defined PM concentration limits as daily and annual mean values which were made legally binding in the European Union. While many studies have focused on PM concentrations in special environments, little is known about the average PM- exposure for both employees and passengers in the German public transportation system.

**Methods:**

Particulate matter (PM10, PM2.5, PM1) – concentrations were monitored for 30 minutes at 15 different areas in Frankfurt am Main with major public traffic. Maximum and mean concentrations and, as a surrogate for the inhaled dosage, the Area Under the Curve (AUC) for 15 minutes of exposure were calculated.

**Results:**

The WHO limits for PM10 and PM2.5 were exceeded at nearly all times and areas. Highest maximum concentrations were found at underground stations, subterranean railway stations and subterranean shopping arcades with much lower values obtained at surface points. In one measurement at a surface test point smokers who neglected the non-smoking policy could be identified as a major cause for a at least temporary strong increase of PM-load as seen in high maximum values and normal averages.

**Conclusions:**

Subterranean areas have high particulate matter contamination exceeding WHO limits. Improvement may be achieved by increased ventilation. Subterranean shops and kiosks, being workplaces with long term exposure, should be equipped with external air supply. The non- smoking policy of the “Deutsche Bahn” for public spaces should be enforced.

## Introduction

Airborne particulate matter (PM) exposure is associated with multiple health risks, such as acute and chronic respiratory problems or increased cardiovascular mortality [1-3]. A considerable part of the air pollution is caused by traffic and may contribute to PH health risks [4]. Many epidemiologic studies have illustrated traffic-associated air pollution and its deleterious effects in children and in adults [5-7]. In recent years, several studies targeted the air quality in railway stations and characterized airborne particulate matter at underground stations, inside underground- and rail wagons and at platforms [8-10].

Physical and chemical properties of particulate matter depend on their origin (e.g. combustion processes, mechanical abrasion, etc.). Mechanically generated PM by abrasions of underground train vehicle chassis, brakes, wheels and rails has shown to cause different effects compared to PM from combustion in urban ambient air [8,11].

The WHO has established limits of PM10 and PM2,5 concentrations which are legally binding in the EU since January 2005 (PM10) and since 2010 (PM2.5) respectively. Mean 24 hour PM10 concentrations must not exceed 50 μg/m^3^, mean annual values 20 μg/m^3^, while PM2.5 concentrations are limited to 10 μg/m^3^ (annual mean) and 25 μg/m^3^ (24 hour) [12]. Since little is known about the burden of PM10, PM2.5 and PM1 from sources like public traffic- and transport systems of a metropolis, we investigated the particulate air pollution in the public transport sector and areas of public commerce in Frankfurt am Main, including two subterranean shopping arcades integrated to underground stations, in a field study. The PM dosage was quantified at 15 areas of major public traffic. Exposure is quantified both as maximum and average PM concentrations.

## Methods

Measurements were taken at 15 areas of major public traffic in Frankfurt am Main in August 2013; all measurements were taken for 30 minutes. The measuring points included

● 2 surface area platforms at the central railway station (“Hauptbahnhof”)

● 3 bus stops at the central railway station (“Hauptbahnhof”) forecourt

● 1 subterranean railway platform (Frankfurt Main Airport)

● 1 surface platform of the Frankfurt interurban train (Dreieich-Offenthal)

● 2 subterranean platforms of the Frankfurt interurban train (“Hauptbahnhof” and “Hauptwache”) sub.

● 2 subterranean platforms of the Frankfurt underground railway system (“Hauptbahnhof” underground and “Hauptwache” underground)

● 3 subterranean shopping arcades linked to large underground stations (2× “Hauptbahnhof”, 30 m and 10 m distance to the underground entrance, 1× “Hauptwache”, 15 m distance to the underground entrance)

● 1 conventional surface shopping center (“MyZeil”)

PM10, PM2.5 and PM1 data were recorded using a portable laser aerosol spectrometer (PLA spectrometer 1.109, Grimm Aerosol Technik, Ainring, Germany) with a 6-seconds data interval time as described for the mobile air quality study (MAQS) in inner cities [13]. Data were immediately transferred to a Lenovo laptop via data logger and stored in Windows-Excel charts. In order to prevent data discrepancies caused by strong winds as previously seen in MAQS, all surface measurements were carried out on calm days in August 2013. Statistical evaluation was performed using GraphPAD Prism 5. Maximum- und mean- PM10, PM2.5 and PM1 concentrations [μg/m^3^] were calculated for each measuring point, the area under the curve (AUC) was calculated for a period of 15 minutes [in μg/m^3^ × s].

## Results

The highest average PM burden was documented at underground stations, followed by the subterranean stations of interurban- and railway trains (Table 1). Surprisingly, mean concentrations and AUC (15 minutes) of PM10, PM2.5 and PM1 at subterranean shopping arcades with access to underground stations clearly exceled any values found at surface railway stations, the central railway station forecourt bus stop or a conventional surface shopping arcade such as MyZeil. The amounts of PM detected in subterranean shopping malls proved to increase with their vicinity towards the next subway station entrance. The lowest mean concentrations and AUC were found at surface railway- and interurban train stations.

**Table 1:** **Maximum and mean concentration of PM10, PM2.5 and PM1 (μg/m**^**3**^**)**

**Place**	**Max. PM**_**10**_	**Max. PM**_**2.5**_	**Max. PM**_**1**_	**Mean PM**_**10**_	**Mean PM**_**2.5**_	**Mean PM**_**1**_
Central Station. Pl. 13	163	120	109	40	28	22
Central Station Pl. 3	235	81	75	44	28	22
Dreieich-Offenthal	567	549	545	46	31	28
Frankfurt Airport	213	88	54	74	46	31
Central Station Sub.	138	71	30	62	33	18
Hauptwache/Sub.	140	68	31	83	53	27
Underground Central Station	166	79	31	92	52	24
Underground Hauptwache	166	85	35	109	65	29
Shopping arcade central station 1	121	57	43	44	24	17
Shopping arcade Hauptwache	97	47	26	61	31	21
Shopping arcade central station 2	324	63	54	73	38	31
Central Station Forecourt 1	135	127	119	35	22	16
Central Station Forecourt 2	212	206	201	37	23	19
Central Station Forecourt 3	230	120	113	50	27	21
Myzeil (Shopping Center)	201	46	32	51	30	26

The unexpectedly high maximum concentration of PM10, PM2.5 and PM1 at the surface interurban train station “Dreieich-Offenthal” was due to cigarette smoke from two passengers standing close to our measuring device for several minutes. Notably, all subterranean points show much higher AUC-PM values than any surface measuring point, despite air conditioning- and ventilation at all subterranean shopping arcades and underground stations. The observed outlying maximum levels of PM with a lower effect on the AUC (e.g. Dreieich-Offenthal, Table 1) are mostly caused by smokers who ignored the non- smoking policy, or were caused by a tourist bus waiting with running engine (central railway station forecourt, Table 1).

Table 1 summarizes the maximum- and mean PM10, PM2.5 and PM1 concentrations of each measuring point.

**Figure 1:**
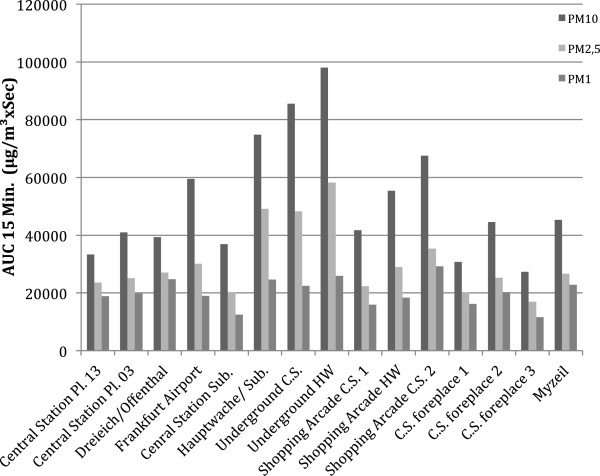
**AUC of the PM10-, PM2.5- and PM1- load at each measuring point.** The AUC was calculated for a duration of 15 minutes assumed as a realistic time for passengers changing connections.

Figure 1 illustrates the relation between PM10, PM2.5 and PM1 AUC values at each measuring point. The dose for waiting, traveling or shopping persons at each of the locations was calculated as “Area Under the Curve” (AUC); for comparison an average of 15 minutes was assumed for people changing trains. As can be seen the PM composition at these areas was similar, with slight differences between the PM fractions. Whereas the highest AUC for PM10 coincided with the PM2.5 value the highest PM1 value was found in a shopping arcade. Also the large variation for PM10 values is not mirrored in PM1 values and only partially preserved in PM2.5 values (Figure 1, Table 2).

**Table 2:** **AUC of PM10, PM2.5 and PM1 (μg/m**^**3**^ **× sec.) calculated for each measuring point referring to 15 minutes**

**Ort**	**PM**_**10**_	**PM**_**2.5**_	**PM**_**1**_
Central Station. Pl. 13	33274	23579	18824
Central Station Pl. 3	40958	25157	19825
Dreieich-Offenthal	39353	27070	24767
Frankfurt Airport	59460	30071	18970
Central Station Sub.	36927	20351	12480
Hauptwache/Sub.	74816	49112	24686
Underground Central Station	85582	48295	22439
Underground Hauptwache	**98025**	**58249**	**25894**
Shopping arcade central station 1	41805	22342	15961
Shopping arcade Hauptwache	55400	29026	18310
Shopping arcade central station 2	67485	35413	**29243**
Central Station Forecourt 1	30756	20099	16240
Central Station Forecourt 2	44610	25223	20325
Central Station Forecourt 3	27363	16976	11626
Myzeil (Shopping Center)	45277	26633	22868

The data in bold represent the highest measured data of their group.

Table 2 summarizes the AUC-data of PM10, PM2.5 and PM1 at each measuring point, calculated for 15 minutes.

## Discussion

In this field study, maximum permissible values for PM10 and PM2.5 as defined by the WHO have been exceeded at nearly all measuring points of public mass transit, when the values are extrapolated to daily or annual mean values. Underground stations, subterranean railway platforms and subterranean shopping arcades have been identified as places with the highest PM- exposure. Although our data were sampling individual places and days, the analysis was executed as a field study. There are no indications that the results were flawed by the time of the day or specific high exposure conditions; thus it is expected that the results can be transferred also to full time employees working under these conditions. Among the clientele with high exposure are e.g. shop assistants in subterranean stations and shopping arcades, cleaning workers or maintenance personnel.

Most of the larger subterranean stations are equipped with their own air supply; however, -our data indicate room for improvement.

## Conclusion

Each shop or kiosk located in a subterranean station should get its independent fresh air supply. From individual measurements it was also evident that smoking is a major cause of PM burden, causing a temporary increase of PM10, PM2.5- and PM1 concentrations. Hence smoking areas should always be designed with sufficient distance to working spaces, and the general non-smoking policy in public areas should be enforced.

## Competing interest

The authors declare that they have no competing interests.

## Authors’ contributions

DAG, JS and AG made substantial contributions to the conception and design of the review, JB and MB acquisred the data and have been involved in drafting and revising the manuscript. All authors have read and approved the final manuscript.
